# Transient Interphase Microtubules Appear in Differentiating Sponge Cells

**DOI:** 10.3390/cells13090736

**Published:** 2024-04-24

**Authors:** Sergei A. Golyshev, Yulia V. Lyupina, Oksana I. Kravchuk, Kirill V. Mikhailov, Nicolay G. Gornostaev, Anton V. Burakov

**Affiliations:** 1 A.N. Belozersky Institute of Physical and Chemical Biology, Lomonosov Moscow State University, Moscow 119992, Russia; sergei.a.golyshev@gmail.com (S.A.G.); kv.mikhailov@belozersky.msu.ru (K.V.M.); 2 N.K. Koltzov Institute of Developmental Biology, Russian Academy of Sciences, Moscow 119334, Russia; yulial@bk.ru (Y.V.L.); kravchuk444@mail.ru (O.I.K.); n_gornostaev@mail.ru (N.G.G.); 3 Kharkevich Institute for Information Transmission Problems, Russian Academy of Sciences, Moscow 127051, Russia

**Keywords:** microtubules, tubulin, evolution, sponges, differentiation

## Abstract

Microtubules are an indispensable component of all eukaryotic cells due to their role in mitotic spindle formation, yet their organization and number can vary greatly in the interphase. The last common ancestor of all eukaryotes already had microtubules and microtubule motor proteins moving along them. Sponges are traditionally regarded as the oldest animal phylum. Their body does not have a clear differentiation into tissues, but it contains several distinguishable cell types. The choanocytes stand out among them and are responsible for creating a flow of water with their flagella and increasing the filtering and feeding efficiency of the sponge. Choanocyte flagella contain microtubules, but thus far, observing a developed system of cytoplasmic microtubules in non-flagellated interphase sponge cells has been mostly unsuccessful. In this work, we combine transcriptomic analysis, immunofluorescence, and electron microscopy with time-lapse recording to demonstrate that microtubules appear in the cytoplasm of sponge cells only when transdifferentiation processes are activated. We conclude that dynamic cytoplasmic microtubules in the cells of sponges are not a persistent but rather a transient structure, associated with cellular plasticity.

## 1. Introduction

Microtubules are the most notable element of the eukaryotic cytoskeleton, named in 1963 by Myron C. Ledbetter and Keith R. Porter [1]. These are highly dynamic tubes with a diameter of 25 nm consisting of polymerized α- and β-tubulin dimers. In interphase cells, microtubules are responsible for the spatial organization of the cytoplasm and intracellular transport [2,3,4,5] for directional cell motility [6,7], as well as for the formation of flagella, cilia, and primary cilia [8,9,10]. Microtubules are also essential for the key process in all eukaryotic cells, forming the mitotic spindle that separates chromosomes during cell division [11,12,13]. The last common ancestor of all eukaryotes already had microtubules and also had dynein and kinesin motors moving along them [14].

Due to their importance, microtubules are conserved in all known eukaryotic organisms. Moreover, the tubulin gene family appears to have emerged very early, perhaps as early as the last common ancestor of all life forms on our planet [15,16]. This is supported by the fact that many prokaryotes have at least one homolog of tubulin, the most common of which is FtsZ. The roles of microtubules in eukaryotes are more diverse than structures formed by tubulin homologs in prokaryotic cells, since they are necessary both for the implementation of mitosis and for the transport of organelles over long distances within much larger eukaryotic cells. The variety of functions performed by microtubules has led to the existence of a wide range of different tubulin isotypes, which determine the appearance of the so-called “multi-tubulin hypothesis” that posits that different specialized tubulins can specify distinct cellular functions [17,18]. In accordance with it, eukaryotes could express unique tubulin isotypes for specific functions, including meiotic spindle assembly in *Drosophila* oocytes [19], axoneme formation in diverse systems [20], and touch receptor neurons in worms [21]. The only example of the opposite situation is the unicellular alga *Chlamydomonas*, which has one α- and one β-tubulin gene that are used for all microtubule functions [22].

The spatial organization of the microtubules in the cytoplasm of interphase eukaryotic cells can be very different; the most typical examples are more or less radial microtubule arrays, columnar microtubule arrays (as in polarized epithelial cells), cortical microtubule arrays in plant cells, and some others (reviewed in [23]). The differences in the microtubule organization in various interphase cells are functionally associated with the different functions of cells within different tissues and directly depend on the activity of microtubule organizing centers, which determine the overall architecture of the tubulin cytoskeleton [24,25,26]. The only two known exceptions to date among all eukaryotes that can successfully dispense with microtubules completely in the interphase are the amoeba *Entamoeba histolytica* [27] and the amoeboid form of another unicellular eukaryote, *Naegleria gruberi* [28,29,30].

Sponges are traditionally considered to be the earliest-diverging animal phylum. Although they do not have differentiated tissues, they possess many types of cells that can be discerned using morphological features [31]. In addition, transcriptomic studies have shown that even more cell types can be identified in sponges using gene expression profiles [32]. The majority of sponge cell types retain a high degree of plasticity and the ability to transdifferentiate [33,34,35]. Such an ability allows sponges not only to heal serious damage but even to reassemble themselves from a suspension of single cells, gradually forming a complete organism with all cell types [36,37]. Recent data suggest that most sponge cells retain such pluripotency [38].

For researchers of the tubulin cytoskeleton, sponges are an extremely interesting object since information about their microtubules is highly inconsistent and mainly refers to the description of choanocytes. Choanocytes are one of the best-described cell types in sponges, owing to their crucial and well-characterized function. They drive water with movements of their flagella, thus dramatically increasing the efficiency of filtration. In addition, the reproductive processes of *Porifera*, due to the lack of gonads in these organisms, show peculiar aspects, as their sexuality takes place through dispersed gametogenesis. The ultrastructural analysis has revealed that in *Spongia officinalis*, male gametes originate from choanocytes [39]. Many articles describe in detail choanocyte flagella, which contain microtubules, and kinetosomes at their bases, which are clearly distinguishable by TEM [40,41,42]. Paradoxically, while the immunofluorescent staining of sponge cross-sections with antibodies to tubulin clearly reveals choanocyte flagella, observations of cytoplasmic microtubule networks in interphase sponge cells have been lacking [35,36]. A recent study has shown that the staining of sponge sections for acetylated tubulin reproduces the pattern of tubulin staining, and staining the specimens of sponges with antibodies specific to acetylated tubulin was used to specifically identify choanocytes among other cell types [43]. On the other hand, there are a number of early studies that have reliably documented the presence of microtubules in other types of interphase sponge cells. Particularly, it was shown that microtubules play a significant role in the active transport of mitochondria in epithelial cells of fresh-water sponges [44,45] and that the microtubules in epithelial cells of *Spongilla lacustris* radiate from the nuclear region and terminate at the cell periphery [46]. However, despite these observations, sponge research is now dominated by the point of view that microtubules and microtubule-based motors are mainly components of flagella. Thus, in another recent paper describing the changes in gene expression during regeneration in explants of the demosponge *Halichondria panacea*, α- and β-tubulins were described as the main structural proteins of the flagellum microtubules, and kinesin/dynein motors were described as important proteins for intraflagellar transport [47].

So, the analysis of published data gives contradictory information about whether the microtubules are present or not in interphase non-flagellated sponge cells. The only indisputable statement we can make is that they must be present in all dividing eukaryotic cells for mitosis to occur. In our study, we investigate the microtubules in *Halisarca dujardini*, which is a common marine demosponge and a filter feeder. We demonstrate that in the absence of cytoplasmic microtubules in interphase sponge cells, they quickly arise when transdifferentiation processes are activated. Our data shed light on the facultative nature of cytoplasmic microtubule networks in early-diverging animals.

## 2. Materials and Methods

### 2.1. Specimen Collection and Cell Cultivation

Specimens of the cold-water sea sponge *H. dujardini* at the period of sponge growth (the end of August and September) were collected from the subtidal zone (0–2 m) at the low tide of the White Sea (66°514′ N, 33°152′ E). The White Sea is a southern inlet of the Barents Sea and part of the Arctic Ocean, located on the northwest coast of Russia. In accordance with local guidelines, permissions for the collection of material were not required. No sponge species were captured in a protected area, national park, or private area, just as no protected or endangered species were involved in the study. The use of sponges in the laboratory does not raise any ethical issues, and therefore approval from regional and local research ethics committees is not required. To induce transdifferentiation, sponges were dissociated and cultivated in seawater filtered with the Millex-GP 0.22 μm filter units (Merck Millipore, Burlington, MA, USA) at an appropriate temperature as described in [48].

Cultured green monkey kidney Vero and COS-7 cells were taken from the lab stocks and grown in DMEM/F12 + GlutaMAX medium (Gibco, Bleiswijk, The Netherlands) supplemented with 7.5% fetal bovine serum (PAA) and penicillin/streptomycin at 37 °C and 5% CO_2_ in the air phase. For primary cilia formation, the cells were starved in serum-free medium for 24 h, as described in [49].

### 2.2. Phylogenetic Analyses

Tubulin and dynein sequences were searched in sponge transcriptomic datasets using BLAST [50]. The sequences for *Halisarca dujardini* were obtained using the transcriptomic datasets deposited under the NCBI BioProject PRJNA594150. The datasets for *Oscarella pearsei* and *Sycon ciliatum* were obtained from the Compagen platform resources (https://compagen.unit.oist.jp/datasets.html (accessed on 15 April 2023)), and the sequences for *Amphimedon queenslandica* and *Homo sapiens* were obtained from the NCBI and UniProt databases. The sequences were aligned with MAFFT version 7 [51] using the L-INS-i algorithm and trimmed for phylogenetic analysis with trimAl [52] using a gap threshold of 0.2. Phylogenetic analysis was carried out by IQ-TREE 2.3.2 [53] using automatic best-fit model selection by ModelFinder [54] and nonparametric bootstrap with 1000 replicates. The trees were visualized using MEGA phylogeny software (version number 7.0.21) [55].

The datasets analyzed during the current study are available in the Sequence reads archive (SRA) under the following numbers: SRR10604734, SRR10604732, SRR10604727, SRR10604726, SRR10604725, and SRR10604724.

### 2.3. Transmission Electron Microscopy (TEM)

For the ultrastructural studies, the sponge fragments and the isolated cells on the coverslips were fixed with a 2.5% glutaraldehyde solution in filtered seawater or (if stated) in 100 mM sodium cacodylate for 24 h. The samples were rinsed three times for 5 min in 100 mM sodium cacodylate and were post-fixed with 1% osmium tetroxide in 100 mM sodium cacodylate for one hour at +4 °C. The samples were dehydrated by increasing the ethanol concentration (50%–70%–96%). Then, 96% ethanol was replaced with acetone, followed by epoxy resin–acetone mixtures with increasing resin content. After replacing the mixture with pure resin (Spi-pon 812, SPI Supplies, West Chester, PA, USA), the resin was cured at +70 °C for 48 h. Coverslips were detached from the resin slabs by the repetitive transfer of the samples from liquid nitrogen to boiling water. Ultrathin sections with a nominal thickness of 80 nm were prepared using a Reichert-Jung Ultracut E ultramicrotome equipped with a Diatome Ultra 45 diamond knife (Reichert, Inc., Depew, NY, USA). The sections were mounted on the formvar-coated copper slot-grids and post-stained with lead citrate for 3 min.

### 2.4. Light Microscopy

Wide-field microscopy was carried out using the Olympus IX71 inverted microscope with an Olympus XM10 14-bit CCD camera equipped with a Micro-Manager software-controlled shutter UniBlitz D122 (Olympus, Tokyo, Japan). Objective lens Olympus PlanApo N 60×/1.42 Oil was used for the immunofluorescence and dry lens Olympus LUC PlanFL N 40×/0.60 Ph2 for lifetime observations. For in vivo observations, the Okolab combined temperature control and humidified CO_2_ system was used in addition to cooling the microscope stage with cold blocks. Briefly, humid ice-cold air passed through a container of water placed in ice, and it was continuously fed into the cooled chamber containing the dish with cells. As a result, the temperature inside the OkoLab incubation box never exceeded +10 °C. We found experimentally that keeping sponge cells at a temperature of 15 °C or above for just ten minutes has a noticeable negative effect on their appearance and motility. Between the observations, the cells were placed in the refrigerator at +6 °C.

For confocal imaging, a Zeiss LSM900 confocal microscope equipped with Zeiss Plan-APOCHROMAT 63×/1.4 Oil DIC objective lens (Zeiss, Oberkochen, Germany) was used (provided by the Moscow State University Development Program).

For immunostaining, the cells were fixed with methanol at −20 °C for 7 min and then post-fixed with 3% paraformaldehyde/PBS at +4 °C for 20 min. Alternatively, the cells were fixed with 3% paraformaldehyde/filtered seawater at +4 °C, and prior to immunostaining, they were permeabilized with 0.5% TritonX100. The following antibodies were used for immunostaining: mouse monoclonal to α-tubulin DM-1A (T9026, Sigma-Aldrich, Saint Louis, MO, USA) and to acetylated tubulin (T6793, Sigma-Aldrich, Saint Louis, MO, USA) and rat monoclonal YOL1/34 to tubulin (Ab6161, Abcam, Cambridge, UK). Species-specific anti-Ig antibodies (MultiLabeling class) conjugated with fluorochromes FITC and TRITC were from Jackson ImmunoResearch Laboratories (UK). The Hoechst staining solution was from Sigma.

### 2.5. Western Blotting Assay

Cell lysates of the *H. dujardini* tissue were extracted in RIPA buffer and protease inhibitor (Sigma). Aliquots containing 80 μg of protein were diluted in sample buffer and maintained for 4 min in a water bath at 95 °C. SDS-PAGE electrophoresis (160 V) in a 12% polyacrylamide gel was then performed, followed by protein transfer to a 0.45 μm nitrocellulose membrane. The membranes were incubated with 5% non-fat milk for 1 h and then incubated overnight at 4 °C with the primary antibodies to α-tubulin DM-1A (T9026, Sigma-Aldrich) or to acetylated tubulin (T6793, Sigma-Aldrich). Incubation for 1.5 h with secondary anti-mouse IgG HRP-conjugated antibodies (GE Healthcare Life Solutions, Boston, MA, USA) was performed, and the blot was immersed in ECL luminol enhancer solution (GE Healthcare Life Solutions, Boston, MA, USA).

## 3. Results

### 3.1. Tubulins and Dyneins Are Highly Conserved from Sponges to Humans

**Figure 1:**
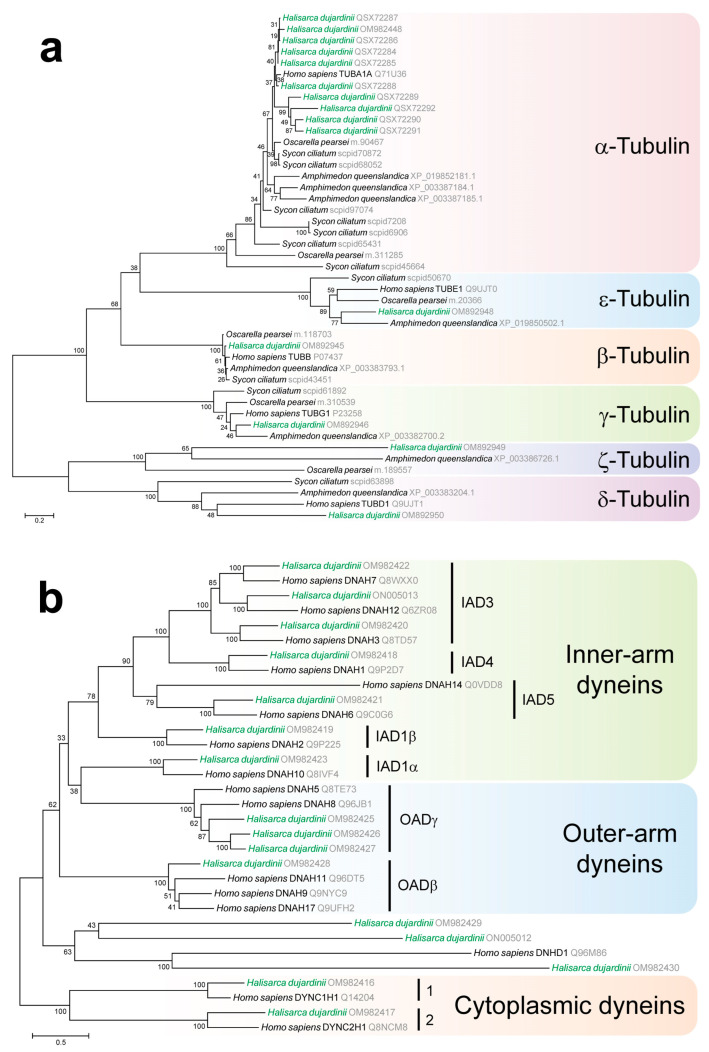
Phylogenetic trees of tubulins and dyneins found in *H. dujardini*. (**a**) A maximum likelihood phylogenetic tree with six tubulin families found in sponges. The tree was reconstructed by IQ-TREE using the best-fitting evolutionary model LG+F+I+G4; the tree includes human tubulin sequences TUBA1A, TUBB, TUBG1, TUBE1, TUBD1, and tubulins found in sponge genomic and transcriptomic datasets: *Halisarca dujardini* (labeled green), *Amphimedon queenslandica*, *Oscarella pearsei*, and *Sycon ciliatum*; (**b**) a maximum likelihood phylogenetic tree with heavy chains of cytoplasmic and axonemal dyneins. The tree was reconstructed by IQ-TREE using the best-fitting evolutionary model LG+F+R5; the tree includes human heavy chain dynein sequences and homologs found in *Halisarca dujardini* (labeled green); branch support for the reconstructed trees was evaluated using nonparametric bootstrap with 1000 replicates; sequence accessions are provided in a lighter font next to the sequence names.

We have searched the transcriptome and the genome of the cold-water sea demosponge *Halisarca dujardini* from the White Sea for tubulins and dyneins to determine whether these proteins are similarly highly conserved in the sponge as in other animals. Similarity searches reveal members of all six tubulin families, conserved across a wide range of eukaryotes, in *H. dujardini* (Figure 1a) (NCBI bioproject PRJNA594150). Namely, the genome of *Halisarca dujardini* contains ten α-tubulin genes and one gene for each of β, γ, ε, δ, and ζ-tubulins. There is also one highly divergent γ-tubulin paralog that is a likely pseudogene. Nine out of ten α-tubulin genes in *H. dujardini* are organized into two clusters in the genome. The genes encoding α- and β-tubulins that make up microtubule protofilaments, as well as other tubulins, including the main factor of microtubule nucleation, γ-tubulin, are highly conserved (see also Table S1). In particular, the sequence of *H. dujardini* α-tubulin is almost identical to that of humans (Figure S1); the greatest similarity to human tubulins is demonstrated by α-tubulin 5 (98.86%). All the a-tubulins of *H. dujardinii* retain conserved nucleotide binding sites and polypeptide binding sites involved in interdimer (β-α) interactions. However, α-tubulins 6–9 have substitutions at conserved sites involved in intradimeric (α-β) interactions (Figure S1).

In accordance with the multi-tubulin hypothesis, the observed diversity of α-tubulins may indicate the wide diversity of functions performed by microtubules in sponge cells. The main function of microtubules in all eukaryotic cells is the formation of the mitotic spindle, and recently, it was shown that the sequences of mitotic tubulins can diverge significantly at key structural positions [56]. The presence of ten different α-tubulins gives grounds to assume the existence of differences not only in the composition of interphase and mitotic microtubules but also in the composition of microtubules deployed by interphase cells. Since sponges are filter feeders and some of their cells have flagella, it is logical to assume the existence of at least two pools of microtubules, strikingly different in their dynamics: cytoplasmic and flagellar. We decided to indirectly prove this through additional transcriptome analysis. The functioning of microtubules is associated with the activity of molecular motors, kinesins and dyneins, which generate forces through ATP hydrolysis. Eukaryotic cells have two diverse groups of dyneins: axonemal dyneins, which provide the motility of the flagellum, and cytoplasmic dyneins, which provide forces for mitotic spindle alignment and positioning during cell division and carry out transport through the cytoplasm during the interphase [57]. Thus, in the context of the multi-tubulin hypothesis, different dyneins move along functionally divergent microtubules consisting of various α-tubulins. We decided to verify the presence of representatives of various groups of dyneins in the sponge. The transcriptome of *H. dujardini* contains key components of the cytoplasmic and axonemal dynein protein complexes and a full suite of conserved dynein heavy chains (Figure 1b). The transcriptome analysis of *H. dujardini* confirmed the gene expression of both axonemal and cytoplasmic dynein subunits (Table S1). Dynein-dependent intracellular transport along the cytoplasmic microtubules usually requires the activity of the multisubunit protein complex dynactin [58], which mediates the attachment of dynein to a cargo and increases the processivity of the cytoplasmic dynein [59]. Transcriptomic data show that all components of the dynactin complex are also conserved in *H. dujardini* (Table S1). It must also be taken into account that the efficiency of intracellular transport often depends on the architecture of the tubulin cytoskeleton, which could depend on the work of microtubule-organizing centers. We conducted additional analysis to find the genes that may be related to the organization of microtubules and confirmed the presence of several key genes (Table S1).

Thus, we observed that sponge cells express genes encoding key proteins involved in the formation of microtubules and proteins that provide intracellular transport along them, as well as proteins involved in the formation and functioning of flagella.

### 3.2. Cytoplasmic Microtubules in Sponge Cross-Sections Are Vanishingly Rare

**Figure 2:**
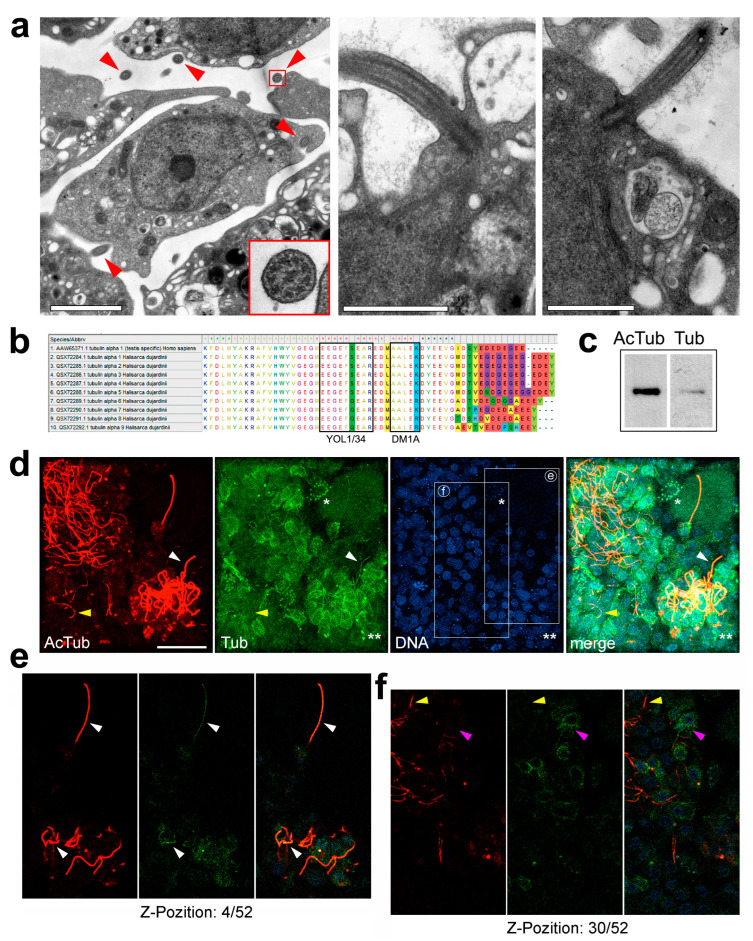
Sponge cross-sections contain mainly flagellar but not cytoplasmic microtubules. (**a**) TEM of ultrathin sections of *Halisarca dujardini*. On the left panel, five flagella cut across are indicated by red arrows, one is also shown enlarged to demonstrate the characteristic internal structure. Scale bar 2 μm. Middle and right panels demonstrate the flagellar microtubules on the longitudinal sections through the flagella that extend from kinetosomes. Scale bars 1 μm. No visible microtubules are present either in the cytoplasm of choanocytes or in the cells without flagella; (**b**) alignments of the amino acid sequence of α-tubulin (tubulin α4a, TUBA4A) of *Homo sapiens* and α-tubulins of *Halisarca dujardini* with MEGA X by Clustal W. The highlighted regions by the frames show the epitopes for YOL1/34 and DM1A antibodies and demonstrate the complete coincidence of these sequences in humans and sponges; (**c**) a Western blot of *Halisarca dujardini* lysates stained with antibodies to tubulin and acetylated tubulin demonstrates the ability of the corresponding antibodies to bind to their antigens in sponge lysates; (**d**) a summarized projection of the sponge cross-section stained with antibodies to acetylated tubulin (red) and tubulin (green). Chromatin stained by Hoechst solution (blue). Z-stack contains 52 planes. White arrows indicate staining for acetylated tubulin coinciding with staining for tubulin, while yellow arrows indicate staining for acetylated tubulin without co-staining for tubulin. The asterisk indicates granules stained for both tubulin and chromatin. Two asterisks indicate tubulin-positive granules that are not stained for chromatin. Rectangles outline the fields of view presented in panels e and f, respectively. Bar, 20 μm; (**e**,**f**) single focal planes from Z-stack shown in panel (**d**); the corresponding Z-positions are indicated below. White and yellow arrows are similar to those in panel (**d**). Magenta arrows indicate staining for tubulin without co-staining for acetylated tubulin. Presumably, such a staining marks dynamic cytoplasmic rather than stabilized flagellar microtubules.

Having confirmed the expression of tubulin genes in the sponge, we then proceeded to visualize microtubules in the sponge cells. The TEM analysis of ultrathin sections of the sponge body shows that it contains cells of different types and is permeated with numerous flagella of choanocytes (Figure 2a). A careful analysis of a large number of TEM images of sponge tissue did not reveal the presence of cytoplasmic microtubules anywhere nor the presence of centrosomes/kinetosomes anywhere except at the base of the flagella. However, it is well known that using TEM makes it easy to confirm the presence of an object, but this method is untenable for justifying the opposite. So, next, we used immunofluorescent staining to examine sponge cross-sections for microtubules.

To exhaustively visualize all microtubules in the sponge, we used two different anti-tubulin antibodies developed in mouse and rat and the mouse antibody against acetylated tubulin for single and double staining. It was dictated by the intriguing results obtained by other authors in several previous studies that demonstrated the identical distribution patterns of tubulin and acetylated tubulin, revealing the choanocyte flagella only in sponge cross-sections [32,35,36,43]. The full coincidence of the tubulin and acetylated tubulin distribution is very atypical, since acetylated microtubules usually constitute only a lesser fraction of the total number of microtubules. Acetylation is one of the post-translational modifications of tubulin, along with detyrosination and polyglutamylation, which preferentially modify microtubules over soluble tubulin dimers [60]. Flagellar microtubules differ in properties from cytoplasmic ones and are stabilized by acetylation, which contributes to their stability and flexibility. We made an alignment of the end region of the α-tubulin sequences of the sponge and human orthologs to make sure that the epitope regions of tubulin are conserved, and we can use the selected antibodies for studying sponges (Figure 2b and Figure S1). The same anti-α-tubulin antibody, clone DM1A, was previously successfully used to detect microtubules in the flagellate form of *Naegleria gruberi*, a single-celled eukaryote that diverged from the “yeast-to-human” lineage over a billion years ago [56]. Prior to the immunostaining of the cells, we performed Western blotting and stained a sponge tissue lysate with antibodies to tubulin and acetylated tubulin. Both antibodies show a clear single band of the expected molecular weight (Figure 2c). We noticed that the staining of acetylated tubulin was much stronger.

We found that choanocyte flagella detected by antibodies to acetylated tubulin are clearly visible in sponge cross-sections, in strict accordance with previously published results (Figure 2d). At first glance, the stain for tubulin repeats this stain in a slightly faded version: the clusters of choanocytes with flagella are visible with double staining for tubulin and acetylated tubulin. Thus, antibodies to tubulin mainly stain flagellar microtubules only, just like antibodies to acetylated tubulin, as previously stated [35,36]. At the same time, dynamic (i.e., non-acetylated) microtubules, which were expected to fill the cytoplasm of all cells in the cross-section, were not visible at all. While creating the summarizing projections, certain background fluorescence is observed in the DM1A channel, which is not at all similar to a set of filamentous structures that could be microtubules. A certain proportion of cells contained DM1A-positive dots, which may correspond to some endosymbionts that are found in abundance in sponge cells (indicated by an asterisk in Figure 2d). Other DM1A-positive granules were not stained with Hoechst solution (indicated by two asterisks in Figure 2d). In any case, all filament-like structures stained with DM1A turned out to be choanocyte flagella, while some of the flagella were stained with antibodies to acetylated tubulin only. So, we studied all the obtained Z-stacks in detail, analyzing each focal plane separately, and found some sponge cells in the tissue fragments that clearly showed non-acetylated microtubules in the cytoplasm (magenta arrow in Figure 2f). Such a staining of microtubules for tubulin that did not colocalize with staining for acetylated tubulin (that is, demonstrating the dynamic cytoplasmic microtubules) was detected in 27 out of 3226 analyzed cells, i.e., less than one percent of cells in sponge cross-sections. This is strikingly different from the typical picture observed in the cells of higher animals. For example, the same staining of cultured mammalian cells from African green monkey kidney demonstrates that acetylated tubulin is detected in a portion of the cytoplasmic microtubules, centrosome, primary cilium, and midbody (Figure S2). Thus, we found that sponge interphase cells contain stabilized acetylated microtubules in the flagella, while dynamic cytoplasmic microtubules are indeed vanishingly rare in sponge cross-sections.

### 3.3. Microtubules Appear Abundantly in the Cytoplasm of Interphase Sponge Cells during Transdifferentiation

The obtained results indicate that the vast majority of sponge cells do not contain any cytoplasmic microtubules. This is fully consistent with previously published data [32,35,36,43]. However, this raises two questions. First, the observed expression of cytoplasmic dynein and dynactin in sponge cells (Table S1) is senseless without intracellular transport along the microtubules. Second, and most importantly, during cell division, the sponge cells must undergo significant rearrangements and create dynamic microtubules to form a mitotic spindle. Therefore, we decided to check the presence of microtubules in sponge cells undergoing dramatic changes, which, however, are not associated with entry into mitosis.

**Figure 3:**
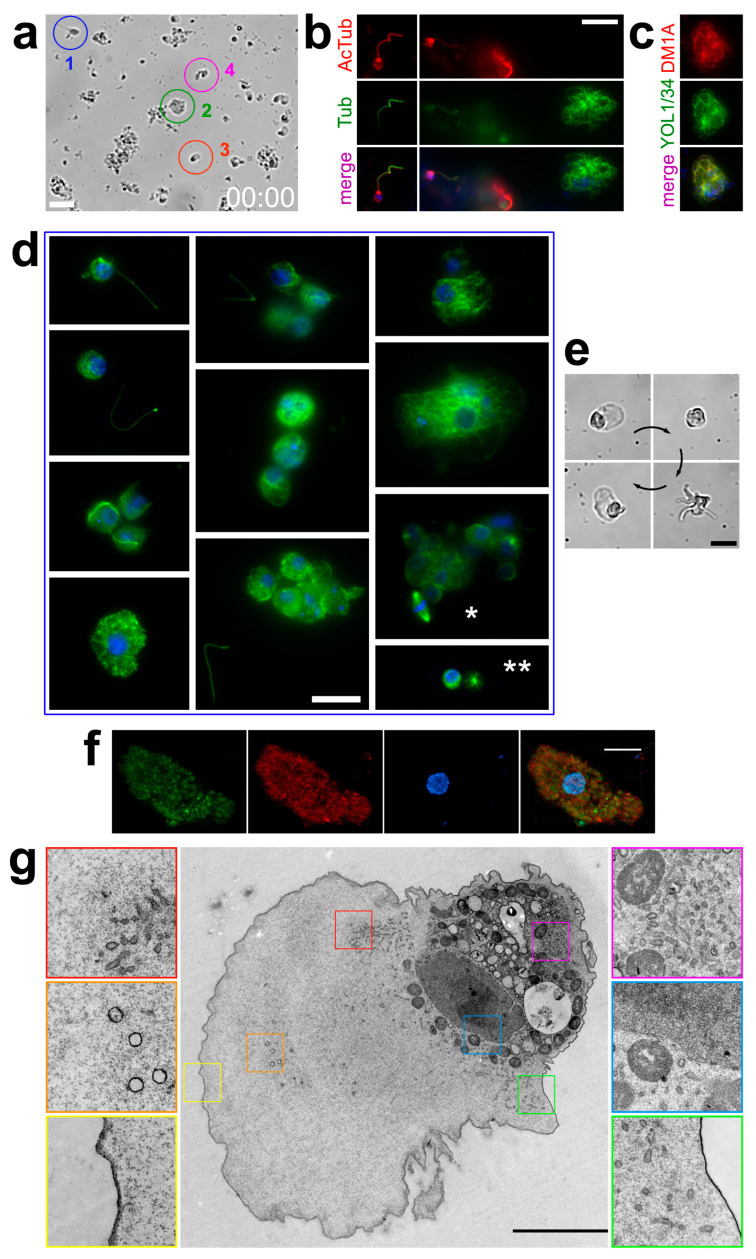
The transdifferentiation of sponge cells is accompanied by the sudden appearance of numerous microtubules in the cytoplasm. (**a**) Typical cell phenotypes in a suspension of sponge cells. This panel represents the first frame of Video S1. Bar, 20 μm. For an explanation of numbers 1–4, see Section 3.3. (**b**) The double immunostaining of sponge cells from suspension with rat antibodies to tubulin (green) and mouse antibodies to acetylated tubulin (red). Blue is chromatin staining by Hoechst solution. The choanocyte on the left demonstrates the same staining as was characteristic in the sponge cross-section in Figure 2d. The example of the spread cell without a flagellum filled with microtubules that do not stain for acetylated tubulin is shown on the right. Bar, 10 μm. (**c**) The immunostaining of single sponge cells with mouse DM1A (red) and rat YOL1/34 (green) antibodies to tubulin and with Hoechst solution (blue). The identical color patterns show the complete colocalization of antibodies which means that microtubule staining with these antibodies is equal to each other. Bar the same as in panel 3b. (**d**) Differentiating sponge cells of various morphologies and sizes staining with antibodies to tubulin DM1A (green) and with Hoechst solution (blue). The non-acetylated microtubules fill the cytoplasm of the cells in abundance. Several flagella broken off from choanocytes, located nearby on the coverslip, are also stained. Note the mitotic spindle staining marked with an asterisk and the cytoplast with a radial aster of microtubules marked with two asterisks. Bar, 10 μm. (**e**) Morphological transformations exhibited by a single spread sponge cell within a few minutes. This panel represents the storyboard of Video S2. Bar, 20 μm. (**f**) Summarized Z-stack of a single spread sponge cell after the end of differentiation processes. Tubulin is shown in green, acetylated tubulin is shown in red, and chromatin is shown in blue. No cytoplasmic microtubules are visible. Bar, 6 μm. (**g**) TEM of an ultrathin section of a single spread sponge cell after transdifferentiation is complete. The individual sections of the cytoplasm containing characteristic organelles are shown enlarged in the insets outlined by lines of the corresponding colors. No cytoplasmic microtubules observed. Bar, 5 μm.

We decided to study the tubulin cytoskeleton of *H. dujardini* during the processes of transdifferentiation, when the dramatic rebuilding of the whole cell architecture occurs. We placed a suspension of sponge cells into a temperature-controlled chamber to observe the early stages of reaggregation in vivo. Cells from the suspension almost immediately start to assemble into small aggregates. Among the single cells on the glass surface, several characteristic morphological types can be easily distinguished, indicated by numbers 1–4 in Figure 3a (see also Video S1). The first type is a choanocyte, free-floating cells moving in the seawater column using a flagellum. The second type is a rather large amoeboid cell that quickly moves on the surface of the glass. The third type is a small amoeboid cell, also moving on the glass very fast. Type 4 cells display a peculiar behavior where the original cell leaves a significant piece of cytoplasm on the glass and, as a result, a small mobile amoeba, similar to those of Type 3, breaks away from it. It is easy to notice a large number of cytoplasmic fragments on the glass, apparently of a similar origin. So, after making sure that we could observe the process of individual cells’ transformation after suspending the sponge, we decided to examine them for the microtubules.

First, we carried out immunofluorescent staining similar to what we performed when studying sponge cross-sections, i.e., mouse antibodies to acetylated tubulin plus rat antibodies to tubulin were used. In choanocytes, the staining repeats the pattern that we observed earlier in sponge cross-sections, that is, the staining for acetylated tubulin and tubulin partially overlap in the flagellum (Figure 3b, left). However, now the immunostaining also reveals numerous cells that were completely absent in the cross-sections, with prominent cytoplasmic microtubule networks that consist of non-acetylated tubulin (Figure 3b, right). The absence of staining for acetylated tubulin in such cells indicates that they contain only newly formed dynamic microtubules. To additionally confirm the quality of cytoplasmic microtubule staining by the YOL1/34 antibodies we used for double immunostaining, we compared it with the mouse monoclonal DM1A stain, the most popular antibody to tubulin. We found that the staining patterns of these two antibodies are fully identical (Figure 3c), which is not surprising given the proximity of their epitopes (Figure 2b). So, we further used both YOL1/34 and DM1A for microtubule staining in sponge cells.

We fixed the sponge cells at various intervals after applying the cell suspension to the coverslips and found that after a brief incubation period, numerous choanocytes cast off their flagella, forming a large number of rounded cells with flagella lying separately on the coverslip. Some cells start to change shape and spread across the glass surface, but many retain a rounded shape and tend to stick together into aggregates. In contrast to the picture observed in sponge cross-sections, these transdifferentiating cells display clearly visible cytoplasmic microtubules (Figure 3d). We also observe mitotic spindles in some of the cells and cytoplasts without a nucleus that contain a radial aster of the microtubules (Figure 3d). So, we demonstrated that after triggering the processes of transdifferentiation by means of sponge dissociation, there is a sharp increase in the number of cells with dynamic microtubules in their cytoplasm. Thus, differentiating sponge cells cease to stand out from other eukaryotes in the absence of microtubules.

Although the sponge cells are pluripotent, the active processes of differentiation are not carried out continuously in the sponge body. Considering that the cells in sponge cross-sections almost do not contain dynamic cytoplasmic microtubules, we decided to check whether the microtubules remain in the cytoplasm of the dissociated sponge cells, which have already carried out the process of differentiation. We removed cell aggregates from the suspension over a period of several days, leaving only the attached cells. As a result, only large amoeboid cells remained in the chamber, which could live up to several months in vitro under proper temperature conditions and the regular replacement of seawater. Lifetime imaging showed that these cells retain motility and easily change shape (Figure 3e, Video S2). They no longer assemble into aggregates, even when they come into contact with each other (Video S3). The double staining of such cells with antibodies to tubulin/acetylated tubulin does not reveal any microtubules, resembling the staining of cells in sponge cross-sections (Figure 3f). Serial TEM ultrathin section analysis also does not reveal microtubules in the cytoplasm of such cells (Figure 3g).

We can conclude that after finishing the transdifferentiation process, the dynamic cytoplasmic microtubules in sponge cells disappear again, and the cells return to their original state, as was observed in the sponge cross-sections. Therefore, the dynamic cytoplasmic microtubules in interphase sponge cells exist exclusively during transdifferentiation processes.

## 4. Discussion

In this work, we studied the microtubules in sponges, where they are typically present as part of the flagella of choanocytes and are not detectable in the cytoplasm of the cells. Based on the obtained results, we can conclude that interphase cytoplasmic microtubules are not obligate but rather facultative structures in sponge cells that are absent outside of the differentiation period and mitosis. Despite the unusual nature of this phenomenon, at least two eukaryotic organisms that lack interphase microtubules were already described before this study. Both of them are unicellular, namely the amoeba *Entamoeba histolytica* and the amoeboid form of *Naegleria gruberi*. The second example is more interesting in the context of the present work because, in contrast to *E. histolytica*, *N. gruberi* has the ability to differentiate from a crawling amoeba to a swimming flagellate [61], which possesses the tubulin cytoskeleton consisting of microtubules and microtubule-binding proteins [62]. The flagellate form is transient, and the cells soon return to the amoeboid form [63], where the microtubules are disassembled. *Naegleria* amoebae lack tubulin transcripts in the interphase [64,65] but do assemble microtubules for closed mitosis [28,29,30,66]. Interestingly, it was recently shown [56] that the expressed mitotic tubulins are unique and are distinct from the tubulins expressed in the flagellate form. While flagellate tubulins are highly similar to tubulins of other eukaryotes, mitotic tubulins have diverged at key residues that are likely to alter microtubule structure and/or dynamics [56]. These data not only support the “multi-tubulin hypothesis” but also serve as a bright example of the fact that the actual presence of microtubules is epigenetically determined at any given moment in eukaryotic cells. However, until now, such phenomena have not been documented in multicellular organisms.

**Figure 4:**
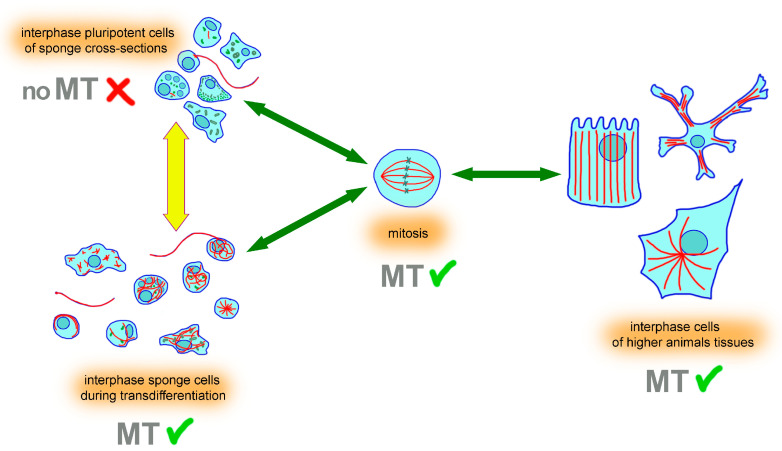
Unlike cells from the tissues of higher organisms, sponge cells contain facultative cytoplasmic microtubules, which exist only during mitosis and transdifferentiation. The scheme summarizes our study and demonstrates the facultative nature of microtubules in sponge cells, where they are present mainly as part of the flagella and also allow for mitosis. This contrasts with the architecture of the tubulin cytoskeleton in the cells of higher animals, where microtubules are present in the cytoplasm both during mitosis and throughout all the interphase. Green arrows indicate a reversible transition from interphase to mitosis, which is accompanied by a reorganization of existing microtubules in cells of higher organisms and differentiating sponge cells or by the arising of microtubules for the formation of a mitotic spindle in sponge cells within the tissue. The yellow arrow indicates the reversible transition of pluripotent sponge cells to the transdifferentiation process, which is accompanied by the appearance of microtubules in the cytoplasm of interphase cells and the subsequent disassembly.

The appearance of cytoplasmic microtubules that we observed clearly explains why tubulin-related machinery is present in sponges, including cytoplasmic dynein and its cofactor dynactin, homologs of such proteins as CLASP 1/2, XMAP215, centrin, and the ancient homolog of pericentrin/AKAP 450 (AKAP9) (Table S1). Apparently, in sponge cells, all these components are just stocked up in an inert state and primed for rapid microtubule assembly upon an activation signal. The nature of this signal, as well as the opposite one, i.e., stimulating the disassembly of microtubules after the end of differentiation or completion of mitosis, remains unknown. Recently, we have already demonstrated the existence of a universal mechanism responsible for the rapid filling of the empty areas of the cytoplasm with microtubules [67]. We showed its work in completely different cells, such as black tetra melanophores and normal human skin fibroblasts, and found that the rapid accumulation of cytoplasmic microtubules was achieved through blocking catastrophes at the plus-ends of microtubules. It is possible that the molecular machinery observed in the cells of vertebrates is a legacy of the archaic regulatory mechanism, which triggers the growth of microtubules throughout the cytoplasm only when needed. The small size of sponge cells presumably absolves them of the need for a developed tubulin cytoskeleton, constantly present to support intracellular transport. The existence of persistent microtubule networks becomes more relevant with the enlargement of the cells, and their architecture would depend on the special cell functions in differentiated tissues, as in fibroblasts, the columnar epithelium, or neurons (Figure 4). Sponge cells, unlike them, are highly pluripotent, and during differentiation, they reversibly build a developed system of microtubules, which then disappear again. Whether this is a consequence of the need to sharply intensify intracellular transport during differentiation is yet to be explored.

The obtained results also raise an interesting question: are the interphase cytoplasmic microtubules needed for transdifferentiation or are they simply a side effect of this process? On the one hand, the rapid repositioning of organelles during transdifferentiation may require a sharp intensification of cytoplasmic transport. On the other hand, the processes associated with post-translational modifications of histones are actively going on in the nucleus during transdifferentiation. It was shown recently by transcriptomic analysis that the expression of histone deacetylase 6 (HDAC6) changes significantly during the dissociation and reaggregation of sponge *H. dujardini* [68]. It is well known that this enzyme is responsible for the deacetylation of tubulin, and the overexpression of HDAC6 increases chemotactic cell motility [69], which may be involved in the process of sponge cell reaggregation. Thus, there is a possibility that the sharp changes in HDAC6 expression could affect the dynamics of the tubulin cytoskeleton. The final answer about the possible active role of interphase microtubules in the processes of differentiation in sponge cells could be obtained by exposing them to appropriate anti-tubulin drugs that stabilize or destabilize microtubules. However, given that some agents that affect microtubule dynamics have been isolated from sponges [70,71], the existence of some mechanisms of resistance to similar inhibitors cannot be ruled out. This will be an interesting direction for future research.

Our work supports the idea that the appearance of the permanent tubulin cytoskeleton as a system of microtubules occurred much later during evolution than the appearance of all the necessary components in the cells, not only did the last common ancestor of all eukaryotes already have microtubules and microtubule motors [14]. Many present prokaryotes have tubulin homologs, the most common of which is FtsZ. This protein is found in both Gram-positive and Gram-negative bacteria, as well as in *Archaea* [72]. In bacteria, FtsZ participates in cell division, forming the Z-ring in the midcell region [73]. In addition, prokaryotic tubulin homologs can form various extended structures; for example, bacterial tubulins A and B (BtubA and BtubB) from *Prosthecobacter dejoneii*, which are closely related to eukaryotic α- and β-tubulins, assemble as a heterodimer [74]. BtubA/B tubular pentagonal structures were found in *Prosthecobacter vanneervenii*; these structures are 200 nm to 1200 nm long and present individually or in bundles of up to four [75]. The four-stranded and two-stranded filament structures were described in *Bacillus thuringiensis* and are assembled from another tubulin homolog, TubZ [76]. Double- or two-stranded TubZ filaments also form bundles when expressed in *E. coli* [77]. Thus, although microtubules with a characteristic complex structure are absent in prokaryotes, bacterial tubulin homologs are already capable of forming composite structures and are involved in vital cellular processes. The role of microtubules that arises in eukaryotes, of course, is much broader, since they are necessary for the implementation of mitosis, intracellular transport, and the functioning of flagella and cilia. The structural components for all this, apparently, were prepared in advance of the emergence of eukaryotes.

From our results, it remains completely unclear what the molecular mechanism is for the appearance and subsequent disappearance of microtubules in sponge cells during differentiation. On the one hand, the regulatory mechanism may be epigenetic, as occurs in unicellular Naegleria, which lacks tubulin transcripts in interphase in the amoeboid state. On the other hand, the ability to rapidly assemble microtubules at sufficiently low temperatures may indicate the presence of powerful mechanisms for regulating microtubule dynamics, in particular, an increase in growth rate or stabilization. This assumption can be supported by the fact that several promising substances were isolated from the sponges, which exert an altering effect on the polymerization dynamics of microtubules [70,71] and could be involved in microtubule assembly. In addition, there is a possibility that some signaling pathways that control microtubule dynamics in animal cells are derived from a common pathway that controlled the emergence or disappearance of facultative interphase microtubules in early eukaryotes.

The ultrastructure of individual microtubules and the basic characteristics of their polar ends, which determine microtubule properties and dynamics, are the same in all eukaryotic organisms, from yeasts and plants to fruit flies and humans. Many aspects of intracellular transport along the microtubule system via motor proteins are also similar in primitive unicellular and higher multicellular organisms. Obviously, the emergence of a complex and highly efficient logistic system, i.e., a microtubule array with molecular motors moving along them, played an important role further in evolution for the origin of highly specialized cells in the differentiated tissues of *Eumetazoa*. But in the small pluripotent cells of sea sponges hundreds of millions of years ago, microtubule asters already flashed briefly during their transdifferentiation.

## Data Availability

All relevant data are within the paper and its Supplementary Materials.

## Supplementary Materials

The following supporting information can be downloaded at https://www.mdpi.com/article/10.3390/cells13090736/s1, Figure S1: Alignments of amino acid sequence of α-tubulin (tubulin α 4a, TUBA4A) of *Homo sapiens* and α-tubulins of *Halisarca dujardini* with MEGA X by Clustal W; Figure S2: The ratio of non-acetylated to acetylated microtubules in cultured mammalian cells; Table S1: Accession numbers for protein sequences of genes in *H. dujardini*, mentioned in the article and accession numbers to their homologs/best blastp hits in *H. sapiens* and *A. queenslandica*; Video S1: Typical cell phenotypes in a suspension of a sponge and the origin of some of them. Time indication: minutes/seconds. Bar, 20 μm; Video S2: Rapid and reversible change in the shape of the single sponge cell. Time indication: minutes/seconds. Bar, 20 μm; Video S3: Spread sponge cells not inclined to aggregates when in contact with each other. Time indication: minutes/seconds. Bar, 20 μm
