# Supplementary material for: Transient Interphase Microtubules Appear in Differentiating Sponge Cells
Source: Cells. 2024 Apr 24;13(9):736. doi: 10.3390/cells13090736 (PMC11082956; doi:10.3390/cells13090736)
Supplement: Supplementary file 1 [file cells-13-00736-s001.zip › Suppl_caption.pdf]

Table S1
Accession numbers for protein sequences of genes in H. dujardinii, mentioned in the article and accession numbers to their homologs / best blastp hits in H. sapiens and A. queenslandica.

Figure S1
Alignments of α-tubulin amino acid sequences of *Homo sapiens* (TUBA3C Hsap) and Halisarca dujardini (TUBA1-10 Hduj) with MEGA X by ClustalW. Amino acids numbering corresponds to human TUBA3C. Highlighted are conservative residues. Nucleotide binding sites are shown with blue triangles, α/β domain interface (polypeptide binding sites) is shown with orange triangles, β/α domain interface (polypeptide binding sites) is shown with gray triangles. Substitutions of conservative residues are shown in gray background.

Figure S2
The ratio of non-acetylated to acetylated microtubules in cultured mammalian cells.

Immunofluorescent staining of African monkey kidney cultured cells, Vero (a,c) and COS-7 (b), with antibodies to tubulin (green) and acetylated tubulin (red). Staining by Hoechst solution is blue. Bar, 10 µm, is the same for a,b,c and d.

(a) The content of acetylated microtubules in neighboring cells may differ when the total number of microtubules is approximately equal
(b) Microtubules in the primary cilium are acetylated, which allows this structure to be visualized in the cells
(c) Acetylated tubulin of stabilized microtubules in midbody which remains after completion of mitosis

Video S1
Typical cell phenotypes in a suspension of a sponge and the origin of some of them. Time indication: minutes:seconds. Bar, 20 µm

Video S2
Rapid and reversible change in the shape of the single sponge cell. Time indication: minutes:seconds. Bar, 20 µm

Video S3
Spreaded sponge cells not inclined to aggregates when in contact with each other. Time indication: minutes:seconds. Bar, 20 µm
