# Supplementary material for: Transient Interphase Microtubules Appear in Differentiating Sponge Cells
Source: Cells. 2024 Apr 24;13(9):736. doi: 10.3390/cells13090736 (PMC11082956; doi:10.3390/cells13090736)
Supplement: Supplementary file 1 [file cells-13-00736-s001.zip › Table S1.pdf]

Supplemental Table 1.

Accession numbers for protein sequences of genes in *H. dujardinii*, mentioned in the article and accession numbers to their homologs / best blastp hits in *H. sapiens* and *A. queenslandica.*

Protein name	*H. dujardinii*	Homologs / best blastp hits			
		H. sapiens		*A. queenslandica*	
	AN	AN	Percent identity	AN	Percent identity
tubulin alpha 1	MT451960	NP_005992.1	96.53%	XP_003387184.1	81.94%
tubulin alpha 2	MT451961	NP_005992.1	96.76%	XP_003387184.1	81.71%
tubulin alpha 3	MT451962	NP_005992.1	95.83%	XP_003387184.1	81.48%
tubulin alpha 4	MT451963	NP_005992.1	96.53%	XP_003387184.1	81.94%
tubulin alpha 5	MT451964	NP_005992.1	98.86%	XP_003387184.1	82.99%
tubulin alpha 6	MT451965	NP_116093.1	86.38%	XP_003387185.1	77.61%
tubulin alpha 7	MT451966	NP_116093.1	86.61%	XP_003387184.1	78.22%
tubulin alpha 8	MT451967	NP_116093.1	87.11%	XP_003387185.1	77.11%
tubulin alpha 9	MT451968	NP_116093.1	79.78%	XP_003387184.1	76.17%
tubulin alpha 10	OM982448	NP_005992.1	95.00%	XP_003387184.1	81.18%
tubulin beta	OM892945	NP_006079.1	99.53%	XP_003383793.1	99.53%
tubulin gamma 1	OM892946	NP_001061.2	82.56%	XP_003382700.2	77.02%
tubulin gamma 2	OM892947	NP_001243821.1	50.92%	XP_003383149.1	55.90%
tubulin epsilon	OM892948	NP_057346.1	62.82%	XP_019850502.1	65.15%
tubulin zeta	OM892949	NP_057345.2	26.24%	XP_003386726.1	28.47%
tubulin delta	OM892950	AAF09584.1	40.60%	XP_003383204.1	39.70%
dynactin 1	OM982405	AAH71583.1	36.92%	XP_003384705.1	41.57%
dynactin 2	OM982406	NP_001248342.1	30.71%	XP_019864101.1	30.16%
dynactin 3	OM982407	no		no	
dynactin 4	OM982408	AAH26323.1	31.16%	XP_003388453.1	29.73%

dynactin 5	OM982409	NP_115875.1	59.66%	XP_003384508.1	58.76%
dynactin 6	OM982410	NP_006562.1	42.93%	XP_003389093.1	47.59%
centrin 1/2	OM982411	NP_004057.1	79.87%	XP_003388306.1	81.88%
centrin 3	OM982412	CAA73077.1	74.67%	XP_003382926.1	69.54%
CKAP5/XMAP215	OM982413	AAI11044.1	49.31%	XP_019850158.1	50.30%
CLASP 1/2	OM982414	XP_016859180.1	35.68%	XP_019850114.1	32.88%
pericentrin/AKAP9 homolog	OM982415	no		XP_019850232.1	35.00%
cytoplasmic dynein 1 heavy chain 1	OM982416	XP_011404630.2	68.93%	NP_001367.2	63.66%
cytoplasmic dynein 2 heavy chain 1	OM982417	XP_003386043.3	53.87%	NP_001368.2	51.14%
axonemal dynein heavy chain 1	OM982418	NP_056327.4	63.02%	XP_019856273.1	70.20%
axonemal dynein light intermediate polypeptide 1	OM982419	NP_003453.3	66.54%	XP_003386434.1	70.99%
axonemal dynein heavy chain 3	OM982420	XP_011544184.1	61.76%	XP_011544184.1	61.76%
axonemal dynein heavy chain 6	OM982421	NP_001361.1	59.46%	XP_011404792.2	69.45%
axonemal dynein heavy chain 7	OM982422	AAL37427.1	64.35%	XP_019854280.1	76.59%
axonemal dynein heavy chain 10	OM982423	NP_001359035.1	60.41%	XP_019853009.1	68.34%
axonemal dynein heavy chain A	OM982425	NP_001193856.1	60.47%	XP_019857825.1	68.02%
axonemal dynein heavy chain B	OM982426	EAX08050.1	60.94%	XP_011403213.1	66.15%
axonemal dynein heavy chain C	OM982427	XP_005248319.2	61.72%	XP_011403213.1	68.54%
axonemal dynein heavy chain D	OM982428	NP_775899.3	62.58%	XP_019853072.1	70.92%

axonemal dynein heavy chain reconstructed 2	OM982429	XP_016865816.1	27.73%	XP_019856560.1	33.97%
dynein-1-beta heavy chain	OM982430	BAG06725.1	22.85%	XP_019859355.1	24.90%
cytoplasmic dynein 1 intermediate chain	OM982431	NP_001258717.1	44.47%	XP_003389902.1	43.47%
cytoplasmic dynein 2 intermediate chain 1	OM982432	NP_001337846.1	31.30%	XP_011405018.2	32.32%
cytoplasmic dynein 2 intermediate chain 2	OM982433	AAH01614.3	36.06%	XP_019859258.1	35.47%
axonemal dynein intermediate chain 1	OM982434	NP_001268357.1	46.25%	XP_019850466.1	47.67%
axonemal dynein intermediate chain 2	OM982435	NP_075462.3	58.62%	XP_019851832.1	64.26%
axonemal dynein intermediate chain 3	OM982436	NP_660155.2	40.83%	XP_019861052.1	43.26%
axonemal dynein intermediate chain 4	OM982437	XP_024305584.1	33.76%	XP_019850930.1	38.90%
dynein axonemal intermediate chain 7	OM982438	NP_001191030.2	22.84%	XP_019855480.1	24.12%
thioredoxin domain-containing protein 3	OM982439	EAW94083.1	36.82%	XP_011403130.2	60.03%
cytoplasmic dynein light chain	OM982440	NP_542408.1	89.89%	XP_003384137.1	87.64%
dynein light chain roadblock-type	OM982441	NP_542408.1	89.89%	XP_003384137.1	87.64%
dynein light chain Tctex-type	OM982442	NP_006510.1	70.80%	XP_003386291.1	72.57%
dynein light chain Tctex-type protein 2B	OM982443	NP_689986.2	61.86%	XP_003389979.1	53.85%
axonemal dynein light chain 1	OM982444	NP_113615.2	67.55%	XP_003384404.1	70.21%

axonemal dynein light chain 4	OM982445	NP_005731.1	65.98%	XP_003386873.1	72.00%
cytoplasmic dynein 1 light intermediate chain	OM982446	XP_016878497.1	42.69%	XP_019858974.1	40.36%
cytoplasmic dynein 2 light intermediate chain	OM982447	BAD97152.1	43.95%	XP_019855996.1	48.97%
axonemal dynein heavy chain E	ON005012	XP_011523718.1	21.98%	XP_019853643.1	31.38%
axonemal dynein heavy chain H	ON005013	NP_001352957.1	60.76%	XP_019850668.1	68.42%
