# Supplementary figures and images for: Transient Interphase Microtubules Appear in Differentiating Sponge Cells
Source: Cells. 2024 Apr 24;13(9):736. doi: 10.3390/cells13090736 (PMC11082956; doi:10.3390/cells13090736)

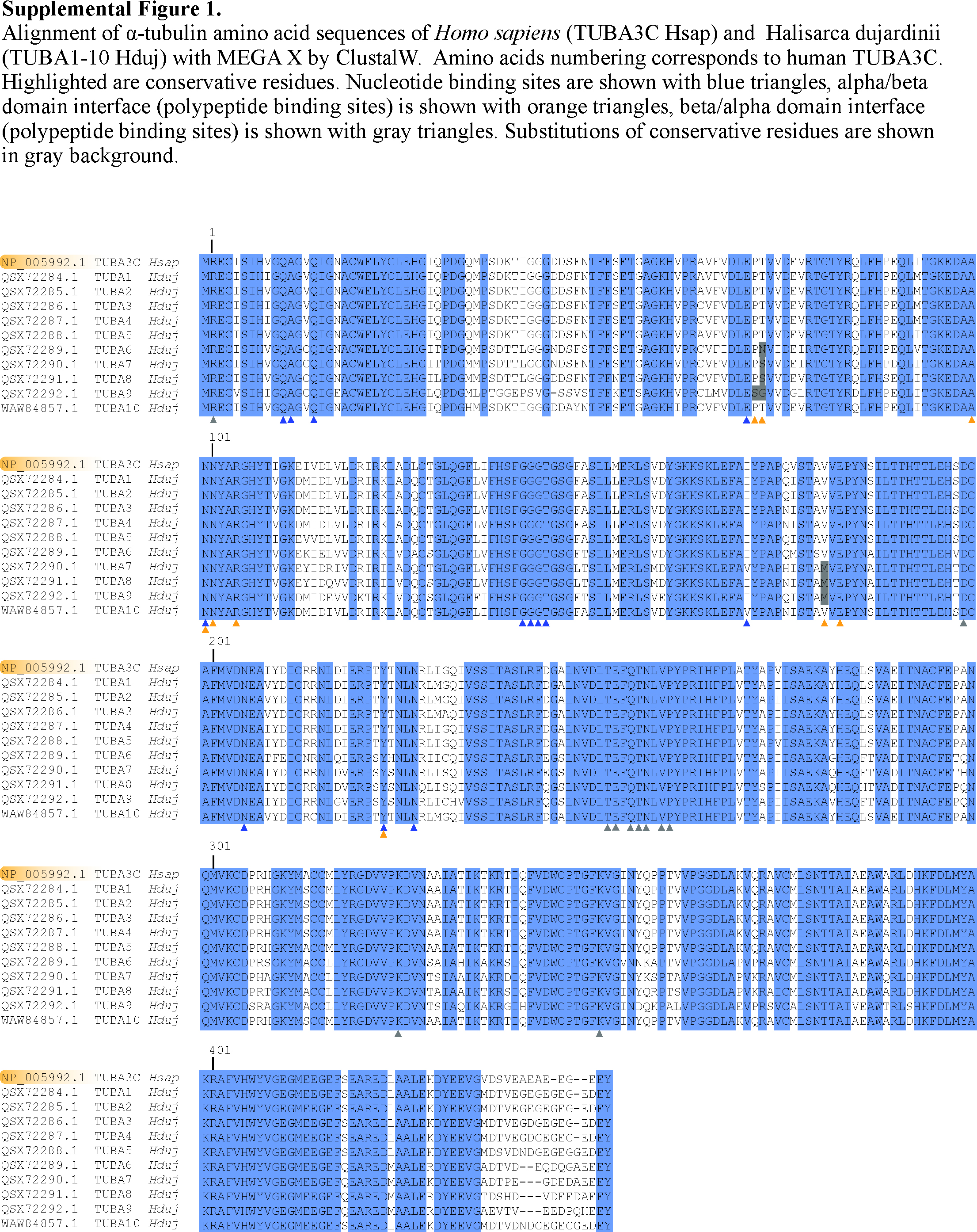

Supplement: Supplementary file 1 [file cells-13-00736-s001.zip › Figure S1.jpg]

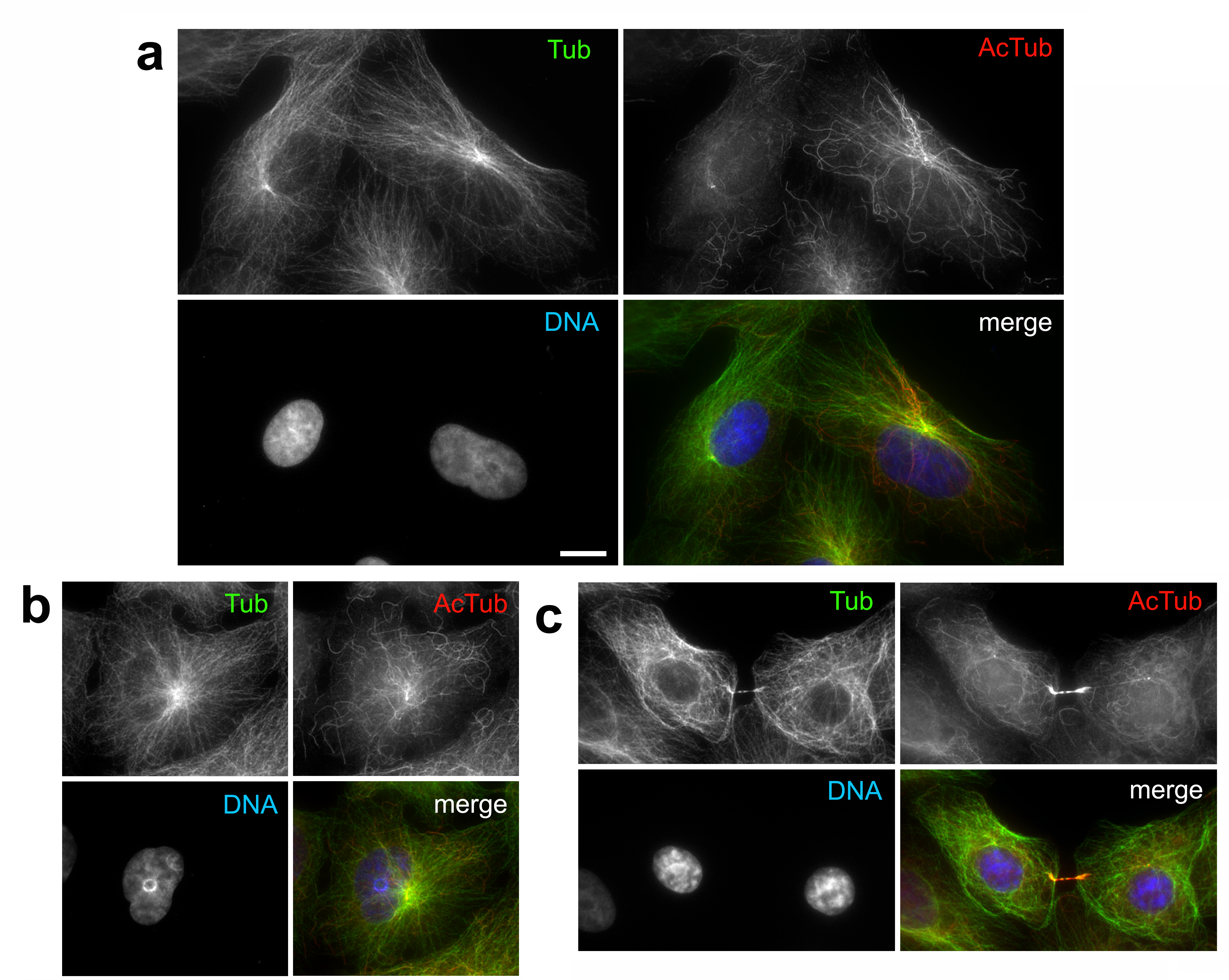

Supplement: Supplementary file 1 [file cells-13-00736-s001.zip › Figure S2.jpg]
